# Survival analysis (Kaplan-Meier curves): a method to predict the future

**DOI:** 10.5935/0004-2749.20200036

**Published:** 2020

**Authors:** Rodrigo Pessoa Cavalcanti Lira, Rosalia Antunes-Foschini, Eduardo Melani Rocha

**Affiliations:** 1 Department of Ophthalmology, Faculty of Medicine, Universidade Federal de Pernambuco, Recife, PE, Brazil; 2 Department of Ophthalmology, Otorhinolaryngology and Head & Neck Surgery, Ribeirão Preto Medical School, Universidade de São Paulo, Ribeirão Preto, SP, Brazil

Survival analysis is a non-parametric statistical model in which the dependent variable is the “time interval” and the independent variable is the “occurrence or not” of a given event^(1-4)^. The analysis measures and compares the lapsed times from interventions or exposures to given “events.” Understanding the concept of median survival, the time interval in which the event occurs in 50% of participants, is essential. The median survival has a similar meaning to that of the means and standard deviation used in classical descriptive statistics. Kaplan-Meier curve estimators are named after the creators of this broadly used method in medicine^(5)^ that estimates the probability of survival, under different conditions and at different time intervals with a graph that illustrates the probability over time.

In medicine, survival analyses are used to predict the longevity of surgical interventions such as fistulas or transplants, as well as the longevity of disease-free or complication-free periods in various forms of chronic diseases and malignancies (Box 1). Survival analyses are excellent for evaluating outcomes and helping to make therapeutic decisions anticipating progressions. These analyses may be applied in comparative longitudinal studies on the safety and efficacy of clinical or surgical treatments.

**Box 1:** Examples of relevant observations in general medicine and ophthalmology, using survival analysis

Subject	Conclusion based on survival analysis	Reference
Prostate cancer 29-year survival	Radical prostatectomy is superior than watchful waiting	Bill-Axelson, Holmberg et al. 2018^(6)^
Prospective investigation to clarify whether revascularization reduces cardiovascular events in patients with heart ischemia	Trial planning to compare invasive versus medical therapy for hearth ischemia, since revascularization over the conservative approach has been questioned in other trials	Maron, Harrington et al. 2018^(7)^
Trabeculectomy outcomes in a tertiary hospital in Brazil	The success rate was 60% after 3 years of surgery	Abe, Shigueoka et al. 2017^(8)^
Autologous corneal limbal transplantation outcomes	The success rate was 77% after 10 years	Rama, Matuska et al. 2010^(9)^
Allogeneic corneal limbal transplantation outcomes	The graft survival rate was 33% after 2 years in patients with Stevens- Johnson syndrome	Santos, Gomes et al. 2005^(10)^

Survival times measure the time interval from a starting point until the occurrence of a given event (e.g., death, cure, relapse, incubation, or equipment failure). Table 1 shows a comparison between the possible outcomes of survival analysis and classical statistics^(3)^ when applied to the same problem. Crucially, survival analysis values the whole curve and not isolated points.

**Table 1:** Comparison between survival analysis and classic statistics

	Survival analysis	Classical statistics
Dependent variable	Time period to event	Occurrence
Association measures	Hazard ratio	Relative risk, odds ratio
Results presentation	Survival table, Kaplan-Meier curve	Table, bar graph, histogram
Test to compare groups for univariate analysis	Log-rank	T-test, ANOVA, KruskalWallis, chi-square
Test for multivariate analysis	Cox regression	Multivariate regression

Adapted from Botelho et al., 2009^(2)^

**Figure 1:**
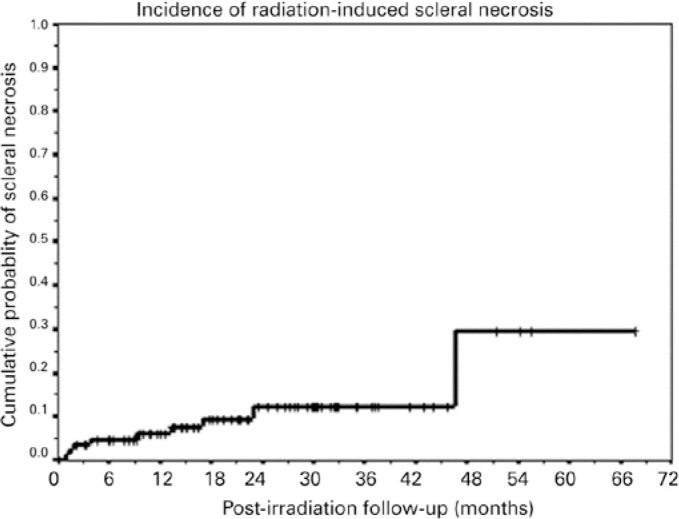
Kaplan-Meier cumulative probability curve showing the incidence of radiation-induced scleral necrosis (SNEC)^(12)^.

Over long follow-up periods, events for some participants may occur before the analysis period. Moreover, many participants get lost to the follow-ups because they leave the study for different reasons (e.g., participants migrate to another city) or the study may end before the event occurs (events may occur after the study period, but research is expensive, and it needs to have a start and a deadline). While in a classic per protocol analysis the above participants get excluded, in survival curve analyses, they are designated as censored^(11)^. If the event happens before the analysis time period, they are censored “on the left,” and if the event does not occur even after the analysis period has ended, they are censored “on the right.” If participants are lost to follow-up in the middle of the study, they constitute the interval censorship (the participant’s time interval until the event cannot be accurately measured). Censored data are represented by crosses or other marks in the graphic presentation (Figure 1). Censorship must follow the basic premise of not being related to prognosis, because if some of the censored individuals in one of the groups have diseases so advanced that they should not be in the study, this will generate a selection bias. Another important assumption is that the prognosis should be the same for all participants, as the characteristics of participants at the beginning and end of the inclusion period must be similar to avoid another selection bias.

One of the advantages of survival analysis is that it considers both data from participants who develop the event and data from those who are censored and can compare survival medians between different groups when the event occurs in at least 50% of the participants.

The comparisons between groups are based on two variables: the survival time interval and the status at the end (occurrence or not of the event or censorship). In the Kaplan-Meier method, the follow-up time is divided into intervals, with limits corresponding to the follow-up time between events, with or without censorship. The likelihood of participants at the beginning of each interval to develop the event by the end of each interval is estimated. Survival at the end of each interval equals the product of cumulative survivals to the end of the previous interval by conditional survival in that interval. Individuals censored in one interval no longer count as individuals at risk in the next interval (Table 2).

**Table 2:** How to calculate survival at each interval

Interval (months)	Numbers of events	Numbers of censures	Number of Individuals at risk	Event probability	Survival probability	Cumulative survival
0-18	1	2	10	1/10	9/10	9/10 = 0.9
19-25	1	1	7	1/7	6/7	0.9^*^6/7 = 0.77
25-36	1	1	5	1/5	4/5	0.77^*^4/5 = 0.62

In survival analyses, groups are compared using some tests: In the log-rank test, the null hypothesis (H0) is that no difference between the survival curves of the two groups exists. If the test shows a P-value <0.05, H0 is rejected and the alternative hypothesis (H1) is accepted, assuming a statistical difference between the groups. Confidence intervals can also be calculated. Hazard is the probability that any participant, who did not have the event, will have it at that time. The hazard ratio compares the instantaneous incidence of events in the different groups and is estimated by the Cox regression test. The ratio estimates the relative risk of developing a particular event within a time interval. The Cox’s proportional hazards are used when the aim is to analyze predictive factors (covariates) that may be interfering with survival.

Care should be taken when interpreting the survival curve in relation to the sample size. For example, from the first censored participant, the survival curve becomes an estimate, so estimates in the final observation periods are quite inaccurate due to the reduction in the number of participants. Another crucial point is proportionality, that is, hazards must be proportional at different time intervals. If the percentage of censorship is asymmetric, caution should be exercised when interpreting results. For example, when comparing two types of cancer treatment, one treatment may have more events (deaths) at the beginning but longer survivals at the end, while the other may progress with better survival at the beginning and more events at the end, showing a disproportionality of events over time, for both groups.

Survival analyses are good for analyzing data behavior over time, the Kaplan-Meier curve estimates the survival curve (descriptive statistics), the log-rank test compares two survival curves, the hazard ratio is a mea sure of association (similar to relative risk), and the Cox regression allows analysis of the influence of predictive factors (covariates). Thus, survival analyses are one of the most scientifically accurate methods to preview the future among all the imperfect approaches.
